# Pattern changes of EEG oscillations and BOLD signals associated with temporal lobe epilepsy as revealed by a working memory task

**DOI:** 10.1186/1471-2202-15-52

**Published:** 2014-04-25

**Authors:** Helka FB Ozelo, Andréa Alessio, Maurício S Sercheli, Elizabeth Bilevicius, Tatiane Pedro, Fabrício RS Pereira, Jane M Rondina, Benito P Damasceno, Fernando Cendes, Roberto JM Covolan

**Affiliations:** 1 Neurophysics Group, Gleb Wataghin Physics Institute, University of Campinas, Unicamp, Campinas, Brazil; 2 Neuroimaging Laboratory, School of Medical Sciences, University of Campinas, Unicamp, Campinas, Brazil; 3 Department of Natural Sciences, Mathematics and Education, Federal University of São Carlos, UFSCar, Araras, Brazil; 4 Centre Hospitalier Régional Universitaire, Université Montpellier 1, Montpellier, França; 5 Sobbel Department of Motor Neuroscience and Movement Disorders, Institute of Neurology, University College London, London, UK; 6 Universidade Estadual de Campinas, Instituto de Física "Gleb Wataghin". Rua Sérgio Buarque de Holanda, 777 - Cidade Universitária Zeferino Vaz, Campinas, SP CEP 13083-859, Brazil

**Keywords:** EEG, fMRI, Alpha and theta rhythms, Working memory, Temporal lobe epilepsy

## Abstract

**Background:**

It is known that the abnormal neural activity in epilepsy may be associated to the reorganization of neural circuits and brain plasticity in various ways. On that basis, we hypothesized that changes in neuronal circuitry due to epilepsy could lead to measurable variations in patterns of both EEG and BOLD signals in patients performing some cognitive task as compared to what would be obtained in normal condition. Thus, the aim of this study was to compare the cerebral areas involved in EEG oscillations versus fMRI signal patterns during a working memory (WM) task in normal controls and patients with refractory mesial temporal lobe epilepsy (MTLE) associated with hippocampal sclerosis (HS). The study included six patients with left MTLE-HS (left-HS group) and seven normal controls (control group) matched to the patients by age and educational level, both groups undergoing a blocked design paradigm based on Sternberg test during separated EEG and fMRI sessions. This test consisted of encoding and maintenance of a variable number of consonant letters on WM.

**Results:**

EEG analysis for the encoding period revealed the presence of theta and alpha oscillations in the frontal and parietal areas, respectively. Likewise, fMRI showed the co-occurrence of positive and negative BOLD signals in both brain regions. As for the maintenance period, whereas EEG analysis revealed disappearance of theta oscillation, fMRI showed decrease of positive BOLD in frontal area and increase of negative BOLD in the posterior part of the brain.

**Conclusions:**

Generally speaking, these patterns of electrophysiological and hemodynamic signals were observed for both control and left-HS groups. However, the data also revealed remarkable differences between these groups that are consistent with the hypothesis of reorganization of brain circuitry associated with epilepsy.

## Background

The electroencephalogram (EEG) has been used since 1930’s decade to monitor, measure and record electrical activity in patients with neurological disorders, especially epileptic discharges in patients with epilepsy and seizure disorders [1,2]. Since then, its applications have been also extended to the study of cognitive functions, which are tested by means of evoked potentials generated in response to external stimulation [3]. One of the most important attributes of this technique is its high temporal resolution (<1 ms) [4], in opposition to its three main limitations: (1) low spatial resolution compromising the precise location of the electrical oscillations origin in the brain, (2) low sensitivity due to its restriction to the more superficial layers of the cerebral cortex, and (3) the requirement of a large cortical area to be simultaneously activated in order to generate enough potential that can be registered by the scalp electrodes [5].

On the other hand, the functional magnetic resonance imaging (fMRI) has been explored since the early 1990s to identify hemodynamic response related to neural activity in the brain, not only of normal controls but also of neurological patients, during resting-state or motor, visual and cognitive tasks [6-10]. One of the most relevant advantages of this neuroimaging technique is its high spatial resolution (~1 mm), which allows the detection of activated regions even in deep cerebral structures (e.g. medial temporal lobe structures) [11]. In contrast, the most relevant disadvantages are (1) its indirect results that are based on the increase of hemodynamic response in certain cerebral areas during determined period or task, which is interpreted as an indirect measurement of neural activity, and (2) its low temporal resolution, which implicates in a delay between the stimulus presentation and the increase of oxygen consumption and, consequently, cerebral blood flow in the cerebral regions involved in a specific period or task [10,12].

More recently, EEG and fMRI have been used in simultaneous or separated acquisitions [13-18], in order to optimize both temporal and spatial resolutions, and to establish possible correlations between electrophysiological and hemodynamic signals. However, it is important to highlight that the simultaneous acquisition requires hardware compatibility and specific software for artifact removal [19-21].

Only a few studies have aimed to investigate similarities, as well as differences, between EEG and fMRI data obtained during a working memory (WM) task in normal controls [15,17,22,23]. When it comes to long-term memory (LTM), however, it is well-established in the literature the relationship between LTM deficits, hippocampal sclerosis (HS), and refractory mesial temporal lobe epilepsy (MTLE), while the consequences of MTLE and HS on working memory (WM) remain unclear. MTLE has been considered to compromise LTM but not WM [24]. However, recent evidence suggests WM is also impaired [25-27], with WM being secondarily disrupted by propagation of epileptic activity from the epileptogenic zone to eloquent cortex responsible for WM function [28], or primarily affected by dysfunction of medial temporal lobe (MTL) structures, mainly the hippocampus, critically involved in WM processes [29,30]. Former evidence, even for a material-specific role of MTL on WM had been shown by studies of verbal WM in patients with left TLE using transcranial magnetic stimulation over the temporal areas [31], and studies of spatial WM in patients with right hippocampal damage [32]. Therefore, our main motivation for studying WM in MTLE patients with HS is to further investigate the role of MTL in WM processes by comparing the cerebral areas involved in EEG oscillations versus fMRI signals patterns, during a WM task in controls and patients with MTLE associated with HS.

## Methods

Both control and left-HS groups were submitted to a working memory task with verbal content during two separated EEG and fMRI sessions. The EEG session was performed in the morning, while the fMRI session was carried out in the afternoon of the current day.

### Ascertainment of subjects

Seven normal controls (1 man and 6 women; mean age 37.1 ± 9.0 years) and six patients (1 man and 5 women; mean age 35.7 ± 8.1 years) with refractory MTLE were included in this study. For this purpose, all of them signed an Informed Consent approved by the ethics committee of UNICAMP Medical School.

The normal controls had no history of neurological and/or psychiatric disorders, and were matched to the patients by age and educational level. The patients, who were followed at our epilepsy clinic, were previously diagnosed with simple and complex partial seizures of MTL origin on clinical history, left interictal epileptiform discharges exclusively over left mid-inferomesial temporal region on serial EEG recordings, left HA and other signs of unilateral left HS in the absence of any other cerebral lesion elsewhere on MRI, and verbal LTM deficits on neuropsychological evaluation [33]. Whereas the HA was defined by a MRI volumetric analysis protocol [34], other signs of HS on MRI were evaluated clinically by one of the authors with experience in neuroimaging investigation in epilepsies (F.C.).

### Working memory task

**Figure 1:**
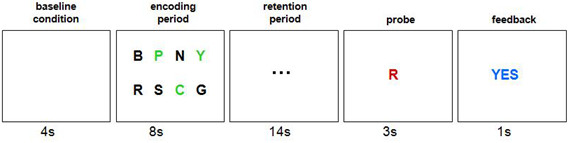
**Working memory paradigm.** Below each frame is the duration of each test block in seconds.

The working memory task was based on the Sternberg test [35]. Each trial began with the presentation of a white screen (4 s), which was followed by the presentation of an array of eight consonant letters (8 s). The individuals were instructed to try to memorize the letters colored black (ranging from three to seven), while ignoring the letters colored green (ranging from five to one, respectively). Soon afterwards, this stimulus array was replaced by an array of three dots (14 s), in which they were requested to try to keep the black letters in their minds. Then, a single probe letter (colored red) was presented (3 s) and they were instructed to press a button only if the letter was among those memorized. Finally, a feedback screen with a “yes” or “no” response (1 s) was presented (Figure 1). Each run consisted of 20 trials (30 s per trial) and was separated from the next one by a two minutes interval, when a black screen was presented. The individuals completed four runs in 46 minutes.

### EEG recordings and analysis

EEG data were recorded separately from fMRI data in order to avoid gradient artifacts, by a 32-channel system (Brain Products, Munich, Germany) referenced to the right mastoid (TP10), at a sampling rate of 1000 Hz with a bandwidth of 0.5 to 70 Hz. Electrodes were positioned using the standard 10-20 system, and their impedances were kept around 5 kΩ. The acquired data were then exported to EEGLAB software (http://www.sccn.ucsd.edu/eeglab/) for preprocessing and frequency analysis. The preprocessing consisted of applying a high-pass filter of 1 Hz to remove eye blink artifacts, and a low-pass filter of 30 Hz to remove muscle artifacts (typically set at 20-50 Hz) and electrical noise (60 Hz). The frequency distribution analyses were separately carried out for the four runs of each subject, and for the two main periods (encoding *versus* retention) of the experiment. For the encoding analysis, one epoch was extracted beginning one second before and ending four seconds after the start of the encoding period. For the retention analysis, three consecutive epochs of four seconds were extracted, leaving aside one second before the first epoch and one second after the last one.

Soon afterwards, all epochs were submitted to extended infomax ICA using *runica* from the EEGLAB toolbox. ICA components with abnormal amplitudes were identified as artifacts and, for this reason, removed [36]. All epochs were then submitted to frequency analysis by FFT decomposition, and the main components were identified and related to expected rhythms.

### fMRI acquisitions and analysis

fMRI images were acquired in a bottom/up interleaved mode, by using a 2 T Elscint Prestige MR scanner with a gradient echo-planar imaging (EPI) protocol (TR = 2000 ms, TE = 45 ms, FOV = 378 × 226 mm^2^, matrix = 128 × 72, in-plane voxel size = 3.0 × 3.0 mm^2^[37], 20 slices no gap, slice thickness = 6 mm, flip angle = 90°). Three hundred and twenty cerebral volumes with 20 slices each were obtained in each run, adding up to four EPI series per subject. The functional images acquired were then (1) reconstructed and temporally reorganized, (2) transformed from DICOM-2D into ANALYZE-3D format, by using the MRIcro software (http://www.sph.sc.edu/comd/rorden/mricro.html), and finally (3) slice timed, realigned, normalized (MNI standard template), smoothed (6 mm/FWHM) and analyzed, by using the SPM software package (http://www.fil.ion.ucl.ac.uk/spm/). Through a Matlab script, it was calculated a mean smoothed image of the four EPI series of each subject and, finally, a mean smoothed image representing each group (control and left-HS groups).

For data analysis, the gamma function was adopted with window length of 32 and order 1 [38-40], as well as the threshold of p = 0.0001 (uncorrected for multiple comparisons) and cluster size of 125 contiguous voxels [41]. For contrast design, we created three conditions [encoding, retention, probe] and assigned the values [1, 0, 0] and [0, 1, 0] to analyze the encoding and retention periods, respectively. The probe condition was not analyzed, but it was designed with null value to not act as baseline condition.

## Results

The two groups were similar as for age (control: M: 37.1/SD: 9.0 years; left-HS: M: 35.7/SD: 8.1 years), educational level (more than 10 years), and correct answers percentage in the WM recognition test (control: M: 91.1/SD: 6.9; left-HS: M: 89.5/SD: 7.2).

### EEG results

Since it was not possible to perform a quantitative group analysis because the EEGLAB software requires time and channels consistency for all individual within the same group, we performed a quantitative individual analysis by using ICA decomposition. These ICA decompositions gave rise to the frequencies values found for each subject of each group. Finally, the frequencies oscillations detected were separated for the two groups. Since the EEG data from two individuals in the control group were too much noisy, they were left out of the final analysis (Additional file 1).

During the encoding period, there was the presence of frontal (mainly in Fz) oscillations ranging from 5 to 7 Hz for both groups, characterizing the theta rhythm. However, it was not possible to discriminate whether these oscillations were originated from frontal or prefrontal structures, due to the limited spatial resolution provided by the 32-channel EEG system used. In addition, there were parietal (mainly in Pz) oscillations ranging from 9 to 10.5 Hz, characterizing the alpha rhythm. These oscillations were more left-sided (P3) in the control group, whereas bilateral without predominance in the patient group. There were also frontal, central and parietal (Fz, F4, Cz, Pz, P3 and P4) frequencies around 17-20 Hz in three patients, characterizing the beta rhythm.

During the retention period, there were alpha oscillations in posterior regions for both control (Pz and P3) and left-HS (Pz, P3 and P4) groups, with absence of theta oscillations in either group.

Finally, these EEG channel locations were related to Brodmann areas [42] (see Additional file 1).

### fMRI results

Additional file 2 shows the cerebral areas of positive and negative BOLD signals in both control and left-HS groups for WM encoding and maintenance periods.

**Figure 2:**
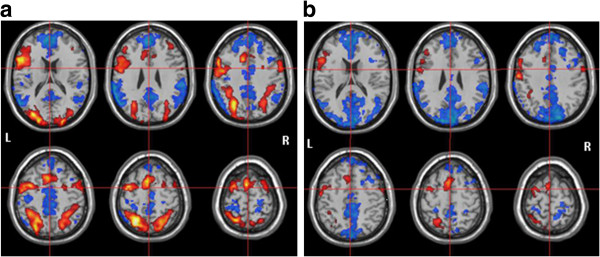
**fMRI results to the control group.** BOLD activation (red) and deactivation (blue) to **(a)** encoding period and **(b)** retention period.

In the control group, data analysis of the WM encoding period revealed positive BOLD in: (1) cingulate gyrus; (2) bilateral > left posterior portion of superior frontal gyrus and medial frontal gyrus; (3) left inferior frontal gyrus; (4) bilateral > left precentral, supramarginal and angular gyrus; (5) left superior parietal lobe, fusiforme gyrus, medial portion of inferior temporal gyrus and insular cortex; (6) bilateral putamen; (7) bilateral lingual gyrus; and (8) bilateral cerebellar cortex. For the WM maintenance, positive BOLD was found in: (1) left superior and medial frontal gyrus; (2) bilateral > left inferior frontal gyrus; (3) left precentral and angular gyrus; (4) left superior parietal lobe; and (5) bilateral cerebellar cortex (Figure 2a).

Moreover, in the control group, data analysis of the WM encoding period showed negative BOLD in: (1) bilateral anterior and posterior portions of cingulate gyrus; (2) bilateral superior frontal gyrus; (3) bilateral > right medial frontal gyrus; (4) right inferior frontal gyrus; (5) bilateral > left supramarginal gyrus; (6) bilateral angular gyrus; (7) bilateral precuneus; (8) bilateral > left superior temporal gyrus; (9) right fusiforme and inferior temporal gyrus; and (10) left insular cortex. For the WM maintenance, negative BOLD was detected in: (1) bilateral anterior and posterior portions of cingulate gyrus; (2) bilateral superior frontal gyrus; (3) right medial frontal gyrus; (4) bilateral supramarginal gyrus; (5) bilateral > right angular gyrus; (6) bilateral superior parietal lobe; (7) bilateral cuneus and precuneus; (8) right inferior temporal gyrus; (9) bilateral lingual gyrus; and (10) bilateral cerebellar cortex (Figure 2b).

**Figure 3:**
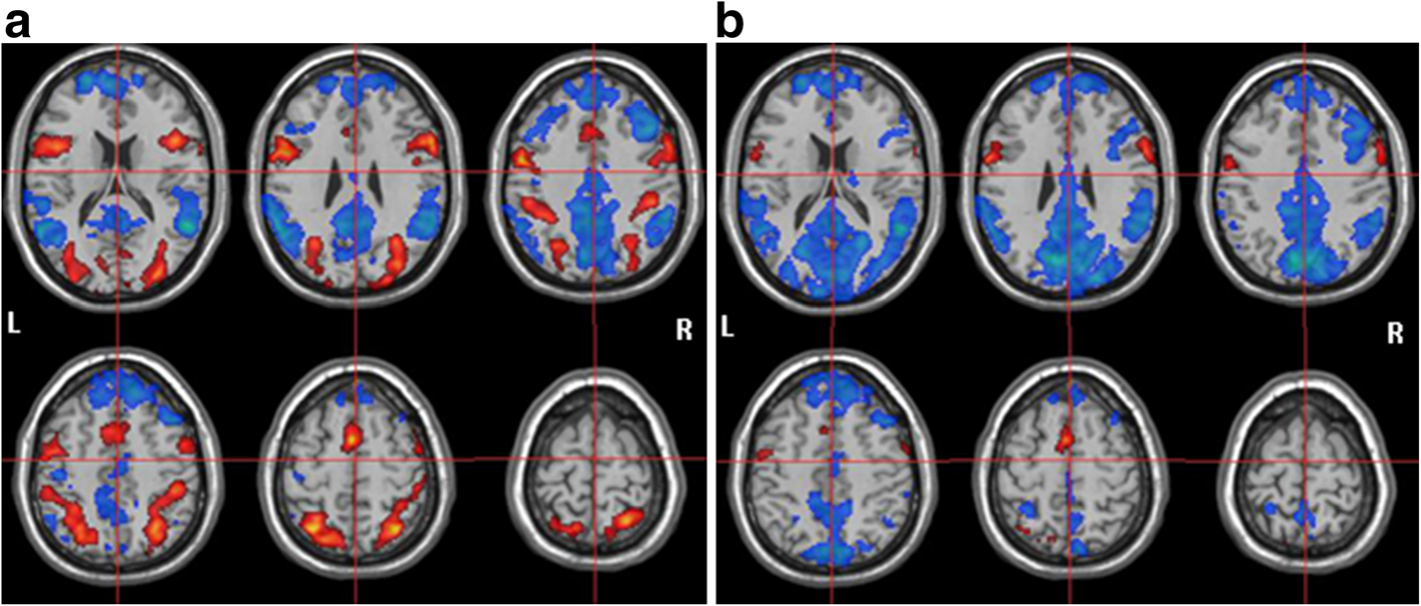
**fMRI results to the left-HS group.** BOLD activation (red) and deactivation (blue) to **(a)** encoding period and **(b)** retention period.

In the left-HS group, the data analysis of the WM encoding period revealed the presence of positive BOLD in: (1) cingulate gyrus; (2) bilateral > left superior frontal gyrus; (3) bilateral > right medial frontal gyrus; (4) bilateral inferior frontal gyrus; (5) bilateral > left precentral gyrus; (6) bilateral supramarginal and angular gyrus; (7) bilateral superior parietal lobe; (8) bilateral lingual gyrus; and (9) bilateral cerebellar cortex. For the WM maintenance, positive BOLD was found in: (1) left superior frontal gyrus; (2) bilateral inferior frontal gyrus; (3) left angular gyrus; (4) left superior parietal lobe; and (5) bilateral cerebellar cortex (Figure 3a).

In addition, in the left-HS group, the data analysis of the WM encoding period showed negative BOLD in: (1) bilateral anterior and posterior portions of cingulate gyrus; (2) bilateral superior frontal gyrus; (3) bilateral > right medial frontal gyrus; (4) left supramarginal gyrus; (5) bilateral angular gyrus; (6) bilateral precuneus; (7) left superior temporal gyrus; and (8) bilateral cerebellar cortex. For the WM maintenance, negative BOLD was detected in: (1) bilateral anterior and posterior portions of cingulate gyrus; (2) bilateral superior frontal gyrus; (3) right medial and inferior frontal gyrus; (4) bilateral supramarginal and angular gyrus; (5) bilateral superior parietal lobe; (6) bilateral cuneus and precuneus; (7) right amygdala; (8) bilateral caudate and internal capsule; (9) bilateral lingual gyrus; and (10) bilateral cerebellar cortex (Figure 3b).

## Discussion

As already presented, there was no significant difference between control and left-HS group with regard to the results of WM recognition test. This finding is in agreement with Tudesco et al. (2010) [43], who did not detect WM deficits in patients with MTLE associated with HS by means of classic neuropsychological evaluation.

### Separated EEG and fMRI data collected in the control group

The EEG results for the control group during the WM encoding period revealed theta oscillations (5-6 Hz) in the frontal area, with peak amplitude in the Fz electrode. This result agrees with some studies in the literature, which have assigned the so-called frontal midline theta rhythm (fmθ) to possible dipolar sources located in the dorsal portion of the anterior cingulated cortex and in the medial prefrontal cortex [15,44-46], and related it to the performance on cognitive tasks that requires a high level of attention, such as WM tests [44,45,47]. Other studies have also associated this increase in theta oscillations in frontal region with the increase in working memory load [15,17,44], emphasizing the preponderant role of frontal lobe structures to the WM encoding and maintenance processes. However, it is important to highlight that we just analyzed the presence or absence of oscillatory rhythms during encoding and maintenance stages, but not the amplitude variations of these rhythms in relation to the amount of items loaded in the WM. On the contrary, WM maintenance did not show theta oscillations, being in disagreement with some other findings in the literature. Some authors detected theta oscillations [48], while other also correlated it to the number of items to be sustained in WM by normal controls [15,17,44].

On the other hand, the EEG results for both WM encoding and maintenance periods demonstrated the presence of alpha oscillations (9-10.5 Hz) in the parietal area, with peak amplitude in the Pz and P3 electrodes, slightly dislocated to the left cerebral hemisphere. According to Gevins et al. (1997) [44], the alpha rhythm is detected not only in the encoding but also in the maintenance stages of WM. However, in opposition to what happens with the theta rhythm, its amplitude tends to decrease with the increase of WM demand.

Although the majority of the studies have focused on the theta and alpha oscillations, some authors have also found delta [49,50] and gamma [51,52] oscillations in normal controls during working memory tasks.

With respect to the fMRI results for the control group during the WM encoding period, a positive BOLD signal was found in the bilateral > left frontal midline area, corresponding to the same region of theta rhythm peak location on the EEG recording. During WM maintenance, a positive BOLD was also detected in the same frontal midline area, although presenting decreased intensity and extension, corresponding to the disappearance of the fmθ on the EEG.

Likewise, a positive BOLD signal was identified in the bilateral > left and left parieto-occipital areas in the WM encoding and maintenance periods, respectively, coinciding with the alpha rhythm location on the EEG. Only in the encoding period, other positive BOLD signal sources were found in the left temporal region, as well as in more medial and deep cerebral structures, but without any corresponding rhythmic activity on the EEG. This lack of oscillatory rhythms in certain cerebral areas is probably due to the EEG low sensitivity to detect electrophysiological signals arising from medial/deep structures, such as putamen, insular cortex, and fusiform and lingual gyrus.

In both periods, there was co-occurrence of positive and negative BOLD signals, which were distributed in adjacent but not coincident cerebral regions. During the WM encoding, the distribution of these positive *versus* negative signals was more counterbalanced in terms of number and extension. Conversely, during the WM maintenance, there was a clear negative signal predominance that remained basically at the same locations, although with larger extension. Nonetheless, there is still a lot of controversy in the literature concerning the relationship between the WM stage (encoding or maintenance) and its associated BOLD signal (positive or negative). Some authors [53,54] have related the maintenance period to the positive signal (“task positive network”), while others [15,17,22,55] have associated it with the negative signal (“default mode network”). Maybe the positive BOLD signals detected during the encoding period are related to the memorizing effort (“task positive network”) of the selected items, whereas the more concentrated and restricted positive BOLD identified during the maintenance period is associated with the sustaining effort (“default mode network”). This sustaining effort may also involve more inhibitory mechanisms, which are expressed by more negative BOLD signals in the fMRI images.

### Separated EEG and fMRI data collected in letf-HS group

In similarity with the control group, left-HS group EEG results revealed the presence of theta oscillations (5-7 Hz) in the frontal area during the WM encoding, but not in the WM maintenance period. Nevertheless, while these theta oscillations were more concentrated in Fz for the controls, they were more distributed between Fz and Cz for the patients during the WM encoding. In addition, similarly to the controls, the EEG results for the left-HS group demonstrated the presence of alpha oscillations (9-10 Hz) in the parietal region (mainly in Pz), during both WM encoding and maintenance periods. However, these alpha oscillations were more extensive and bilateral (P3 and P4) for the patients, whereas they were more left-sided (P3) for the controls. Taken together, these findings indicate a more widespread central theta and bilateral alpha oscillations for the left-HS group, probably to compensate for their left mesial temporal dysfunction.

In the left-HS group, in contrast to the control group, other frequencies oscillations around 17 Hz, 18 Hz and 20 Hz, consistent with the beta rhythm, were also observed in the frontal, central and parietal (Fz, F4, Cz, Pz, P3 and P4) areas in three patients during the WM encoding, and in two of them during the WM maintenance. In other studies using similar WM paradigms (Sternberg task), significant positive correlations between beta/gamma and the BOLD signal have been found more focally than the correlations in lower frequency bands (theta and alpha), which are localized in larger cortical networks, e. g. in the default mode network (DMN) [22]. These local field potential oscillations in the beta/gamma range, highly correlated to the BOLD response, probably represent neuronal assembly dynamics cognitive processing [56]. On the other hand, our finding of other oscillatory rhythms in the left-HS group may indicate a reorganization of the patients´ neuronal network involved in the WM task, as an attempt to overcome the left hippocampal system dysfunction.

As regards fMRI results for the left-HS group during the WM encoding period, a positive BOLD signal was found in the frontal midline area, matching with the frontal midline theta rhythm location on the EEG recording. During the WM maintenance, a decrease of this signal was observed, which was in accordance with the disappearance of the theta oscillations. Other frontal regions also presented positive signals, such as the lateral middle and inferior frontal and precentral gyrus. However, whereas these activations were bilateral > left for the controls, they were bilateral for the patients. Likewise, a positive BOLD was identified in the bilateral and left parieto-occipital areas in the WM encoding and maintenance periods, respectively, coinciding with the alpha rhythm location on the EEG. Nonetheless, in opposition to the controls who exhibited bilateral > left positive signals, the patients showed bilateral positive signals during the encoding period. Conjointly, these findings suggest a more bilateral engagement of frontal and parietal regions, without right or left predominance, or a greater involvement of right frontal and parietal structures for the encoding stage in the left-HS group. Finally, in contrast to controls, no positive BOLD signal was found in the temporal area during WM encoding or maintenance periods in left-HS patients, probably due to their hippocampal lesion.

Similarly to controls, there were: (1) coexistence of positive and negative BOLD signals, which were distributed in adjacent cerebral regions in both WM encoding and maintenance; and (2) the encoding period was characterized by a more counterbalanced distribution of positive *versus* negative signals, while the maintenance period was marked by a clear negative signal predominance. On the other hand, the most important difference between the two groups was that, while controls had exhibited positive and negative BOLD signals in temporal area for both WM encoding and maintenance, the patients had no positive and less negative signals in this cerebral region for encoding and maintenance periods, respectively. In general, these results are in line with other studies [57-60] indicating, from different experimental contexts, network reconfiguration induced by neuronal damage associated with MTLE.

### Limitations

The reduced patient sample may be seen as one of the drawbacks of the present study. Its main purpose, however, is to present evidence of changes in patterns of EEG and fMRI signals that may be associated with brain plasticity due to epilepsy. Certainly, a more extensive study, including right-HS patients, would be needed to further explore this finding. Another limitation comes from the coarse resolution of the EPI images acquired in this study. While some authors [61-63] have found bilateral symmetrical hippocampal activation in normal controls and bilateral-asymmetrical hippocampal activation in MTLE patients, we did not find clear hippocampal activations. This is most likely due to partial volume effects, since in low resolution images nonactivating white matter is averaged with the signal from hippocampal gray matter leading to loss of BOLD signal [62]. Additionally, it should be added a restriction we had on the analysis of the EEG data. The EEGLAB software used in this study did not allow perform a quantitative group analysis, since it requires time and channels consistency for all individuals within the same group, which was not achieved. Thus, a quantitative analysis was performed individually using ICA decomposition, which resulted in values of frequencies found for each subject in each group. Despite this limitation, it was possible to find consistency in the results of individuals in each group.

## Conclusions

EEG and fMRI are brain mapping techniques that rely on different aspects of the neurophysiological processes underlying brain dynamics and whose respective characteristics are interrelated by mechanisms that are still not well understood. For instance, the correlation between EEG rhythms and BOLD-fMRI signal in working memory studies remains a quite controversial subject. The objective of this work was to compare the cerebral areas involved in EEG oscillations versus fMRI signal patterns during a WM task in normal controls and patients with refractory mesial temporal lobe epilepsy associated with hippocampal sclerosis.

For the control group, there was strong correspondence between the theta (Fz) and alpha (Pz and P3) rhythms locations on EEG recordings and the positive and negative BOLD signals on the fMRI. We found: (1) involvement of bilateral > left cerebral areas in both WM periods, and (2) while the encoding period was characterized by the counterbalanced distribution of positive and negative BOLD signals, the maintenance period was marked by the clear negative signal predominance (DMN).

On the other hand, for the left-HS group, there was also a correspondence between the locations of theta (Fz e Cz) and alpha (Pz, P3 e P4) rhythms and the positive and negative BOLD signals. Compared to controls, we observed in the patients group: (1) the same patterns of distribution of BOLD signals for WM encoding and maintenance periods, (2) absence and decreased number of temporal structures with positive and negative signals, respectively, during the encoding period, possible due to the presence of the hippocampal pathology, and (3) engagement of bilateral cerebral regions, without side predominance, emphasizing the role of both left and right cerebral hemispheres for WM processing, probably to compensate for its medial temporal dysfunction.

As a whole, these results reveal that there are characteristic patterns of EEG and fMRI signals generated under working memory task which present peculiar features for healthy subjects and epilepsy patients. These features are specific to each group and possibly indicate differentiation in the neuronal circuitry organization involved in WM function resulting from the pathological condition.

## Abbreviations

WM: Working memory; MTLE: Mesial temporal lobe epilepsy; HS: Hippocampal sclerosis; EEG: Electroencephalogram; fMRI: Functional magnetic resonance imaging; BOLD: Blood oxygenation level dependent; HA: Hippocampal atrophy; MRI: Magnetic resonance imaging; ICA: Independent component analysis; FFT: Fast Fourier transform; EPI: Echo-planar imaging; MNI: Montreal Neurological Institute; SPM: Statistical parametric mapping.

## Competing interests

The authors declare that they have no competing interests.

## Authors’ contributions

HFBO and AA: Conception and design of the work; acquisition, analysis, and interpretation of data for the work; drafting the manuscript. MSS: Substantial contributions to the acquisition and analysis of data for the work. EB, TP and FRSP: Substantial contributions to the acquisition of data for the work. JMR: Substantial contributions to the analysis of data for the work. BPD and RJMC: Drafting the manuscript; revising the work critically for important intellectual content and final approval of the version to be published. FC: Revising the work critically for important intellectual content and final approval of the version to be published. All authors read and approved the final manuscript.

## Supplementary Material

Additional file 1: Table S1 Descriptive analysis of EEG. EEG findings to the normal control and left-HS groups. Click here for file

Additional file 2: Table S2 Descriptive analysis of fMRI. fMRI findings to the normal control and left-HS groups. Click here for file
